# Factor VIII moiety of recombinant Factor VIII Fc fusion protein impacts Fc effector function and CD16^+^ NK cell activation

**DOI:** 10.3389/fimmu.2024.1341013

**Published:** 2024-04-09

**Authors:** H.A. Daniel Lagassé, Jiayi Ou, Zuben E. Sauna, Basil Golding

**Affiliations:** ^1^ Division of Hemostasis, Office of Plasma Protein Therapeutics CMC, Office of Therapeutic Products, Center for Biologics Evaluation and Research, U.S. Food and Drug Administration, Silver Spring, MD, United States; ^2^ Office of Plasma Protein Therapeutics CMC, Office of Therapeutic Products, Center for Biologics Evaluation and Research, U.S. Food and Drug Administration, Silver Spring, MD, United States

**Keywords:** Fc-fusion, Fc gamma receptors, natural killer cells, Fc gamma receptor IIIa, Factor VIII light chain, discoidin domain

## Abstract

Recombinant Factor VIII-Fc fusion protein (rFVIIIFc) is an enhanced half-life therapeutic protein product used for the management of hemophilia A. Recent studies have demonstrated that rFVIIIFc interacts with Fc gamma receptors (FcγR) resulting in the activation or inhibition of various FcγR-expressing immune cells. We previously demonstrated that rFVIIIFc, unlike recombinant Factor IX-Fc (rFIXFc), activates natural killer (NK) cells via Fc-mediated interactions with FcγRIIIA (CD16). Additionally, we showed that rFVIIIFc activated CD16^+^ NK cells to lyse a FVIII-specific B cell clone. Here, we used human NK cell lines and primary NK cells enriched from peripheral blood leukocytes to study the role of the FVIII moiety in rFVIIIFc-mediated NK cell activation. Following overnight incubation of NK cells with rFVIIIFc, cellular activation was assessed by measuring secretion of the inflammatory cytokine IFNγ by ELISA or by cellular degranulation. We show that anti-FVIII, anti-Fc, and anti-CD16 all inhibited indicating that these molecules were involved in rFVIIIFc-mediated NK cell activation. To define which domains of FVIII were involved, we used antibodies that are FVIII domain-specific and demonstrated that blocking FVIII C1 or C2 domain-mediated membrane binding potently inhibited rFVIIIFc-mediated CD16^+^ NK cell activation, while targeting the FVIII heavy chain domains did not. We also show that rFVIIIFc binds CD16 with about five-fold higher affinity than rFIXFc. Based on our results we propose that FVIII light chain-mediated membrane binding results in tethering of the fusion protein to the cell surface, and this, together with increased binding affinity for CD16, allows for Fc-CD16 interactions to proceed, resulting in NK cellular activation. Our working model may explain our previous results where we observed that rFVIIIFc activated NK cells via CD16, whereas rFIXFc did not despite having identical IgG1 Fc domains.

## Introduction

Hemophilia A is an X-linked bleeding disorder caused by a deficiency in functional Factor VIII (FVIII) protein levels. Individuals with severe hemophilia A (plasma FVIII levels < 1%, which represent approximately 60% of patients), require replacement of FVIII to levels sufficient for maintaining hemostasis, and reducing the likelihood of joint bleeding and damage (1). Factor VIII replacement therapy typically includes routine prophylactic dosing levels of Factor VIII products at 20-50 International Units (IU) per kilogram of body weight. Currently, a wide variety of therapeutic products are available for the treatment of hemophilia A patients. These biological products include plasma-derived FVIII concentrates, recombinant FVIII analogs, a bispecific FVIII mimetic, and an adeno-associated virus vector-based gene therapy (2, 3). However, a major shortcoming of many plasma-derived and recombinant FVIII products is the relatively short circulating half-life (~12-15 hours), thus requiring frequent dosing (4). In the past two decades, drug manufacturers have designed novel protein products to overcome FVIII’s intrinsic short plasma FVIII half-life. Such so-called extended half-life (EHL) engineered FVIII products include PEGylated and Fc-fusion protein versions (3). Fc-fusion is a protein engineering strategy used to extend the circulating half-life of a bioactive fusion partner (peptide or protein) via genetic fusion with the immunoglobulin G (IgG) Fc domain which mediates interactions with the neonatal Fc receptor (FcRn) (5–7). Fc binding to FcRn within acidified endosomes avoids intracellular protein catabolism and shuttles the Fc-containing protein into a naturally occurring protein recycling pathway (8). The FcRn-mediated recycling pathway is responsible for the relatively long serum half-lives (~ 21 days) of IgG and albumin.

Antihemophilic Factor (Recombinant), Fc Fusion Protein is a marketed FVIII product for the treatment of hemophilia A. Prior *in vitro* studies have demonstrated that the recombinant Factor VIII-Fc fusion protein (rFVIIIFc) interacts with high-affinity and low-affinity Fc gamma receptors (FcγR) expressed by different immune cell populations (9–13). The immunomodulatory effects of the rFVIIIFc-FcγR interactions differ by the receptor and cell population, but include both activating and inhibitory pathways, similar to canonical IgG1 Fc-FcγR interactions.

We previously demonstrated that rFVIIIFc, unlike rFIXFc, activates natural killer (NK) cells via Fc-mediated interactions with FcγRIIIA (CD16) (12). Furthermore, we demonstrated rFVIIIFc-activated CD16^+^ NK cells lyse an anti-FVIII B cell clone (12). Here, we used human NK cell lines and primary NK cells enriched from peripheral blood leukocytes to further characterize rFVIIIFc-mediated NK cell activation. We identified the FVIII light chain (LC) as playing a key role in the activation of CD16^+^ NK cells by rFVIIIFc. Using antibodies that target different FVIII domains, we demonstrated that blocking C1 or C2 domain-mediated membrane binding. potently inhibited rFVIIIFc-mediated CD16^+^ NK cell activation, while targeting FVIII heavy chain (HC) domains did not. Our results suggest FVIII LC-mediated membrane binding possibly increases binding avidity of Fc to CD16, resulting in NK cellular activation. Moreover, we show that rFVIIIFc binds to CD16 with higher affinity than rFIXFc, and with similar affinity as obinutuzumab, a glyco-engineered (afucosylated) therapeutic IgG1 monoclonal antibody. Typically, low affinity FcγR (i.e., FcγRIIA, FcγRIIB, and FcγRIIIA) activation and functional response requires receptor crosslinking and increased avidity via binding of multiple IgG1 Fc domains with cell surface receptors (14, 15). We propose a working model in which we hypothesize that the LC C1 and C2 domains of the FVIII component of rFVIIIFc bind the NK cell plasma membrane in addition to Fc domain engagement with CD16. Additionally, the FVIII moiety of rFVIIIFc, unlike the FIX moiety of rFIXFc, increases the affinity of the Fc binding to CD16. Together, the FVIII LC- and Fc-mediated interactions result in increased avidity and NK cell activation. Our previous findings and working model provide a plausible explanation for retrospective case reports (16–18) and a prospective clinical study [NCT03093480] (19) suggesting that rFVIIIFc may rapidly tolerize hemophilia A patients with inhibitors (neutralizing anti-drug antibodies) during immune tolerance induction (ITI) therapy. In light of our findings, we also speculate on broader implications for design of novel Fc-fusion proteins to serve as antigen-specific B cell depletion agents.

## Materials and methods

### Source of recombinant proteins and inhibitors

rFVIIIFc, B domain deleted (BDD) rFVIII, and Immune Globulin Intravenous (Human) (IVIG) products were purchased from ASD Healthcare. Rituximab, obinutuzumab, full-length (FL) rFVIII, and rFIX products were purchased from the NIH Pharmacy. rFVIIIFc and rFIXFc were acquired from Biogen through a Material Transfer Agreement. Lyophilized therapeutic protein products were reconstituted with manufacturer-provided diluent per manufacturer’s instructions. All therapeutic proteins were frozen at -80°C as small aliquots and thawed on ice prior to use. The reconstituted drug products were adjusted to the final working concentration using assay buffer.

Anti-FVIII single-chain variable fragment (scFv) (Clone KM33) was kindly provided by Dr. Andrey Sarafanov (FDA/CBER). Anti-FVIII monoclonal antibodies (mAb) GMA-8006, GMA-8013, GMA-8029, GMA-8014, and GMA-8002 were purchased from Green Mountain Antibodies (Burlington, VT). Human von Willebrand Factor (FVIII-free) [# HCVWF-0191] was purchased from Hematologic Technologies (Essex Junction, VT). Recombinant Human IgG1 Fc [# 778308] was purchased from BioLegend (San Diego, CA). Recombinant Human LRP1-GST [# H00004035-P01], Recombinant Human alpha 2-macroglobulin [# 1938-PI], Recombinant Human LRPAP (RAP) [# 4296-LR], Recombinant Human MFG-E8 (Lactadherin) [# 2767-MF] were purchased from Bio-Techne (Minneapolis, MN). Anti-CD16 F(ab’)2 [Clone 3G8] [# 165-520] was purchased from Ancell Corporation (Bayport, MN). Goat anti-human IgG Fc fragment polyclonal antibody [# A80-104A] and goat anti-guinea pig IgG polyclonal antibody [# A60-110A] were purchased from Bethyl Laboratories (Montgomery, TX). Anti-CD16 mIgG1 [Clone 3G8] [# 550383], purified mouse anti-human LRP1/CD91 [# 550495], PE mouse anti-human LRP1/CD91 [# 550497], and mouse IgG1 isotype control [# 550878] were purchased from BD Biosciences (San Jose, CA). Biotinylated anti-FVIII mAb was generated using mouse anti-human FVIII IgG [Hematologic Technologies # AHVIII-5025] and the EZ Link Sulfo-NHS-LC biontinylation kit [ThermoFisher # 21435].

### Human NK cell lines [NK-92 and PTA-6967]

Human NK cell lines, NK-92 (ATCC #CRL-2407) and PTA-6967 (ATCC #PTA-6967) were maintained in NK cell media as described previously (12). NK cell media [Alpha Minimum Essential medium without ribonucleosides and deoxyribonucleosides but with 2 mM L-glutamine and 1.5 g/L sodium bicarbonate (Gibco #12561-056) was supplemented with 0.2 mM inositol, 0.1 mM 2-mercaptoethanol, 0.02 mM folic acid, 12.5% horse serum (Gibco #16050-122), 12.5% fetal calf serum (Gibco #10438-034), and 100 U/mL recombinant IL-2 (R&D Systems #202-IL)].

### Isolation and culture of primary NK cells from PBMCs

Commercially available cryopreserved peripheral blood mononuclear cells (PBMCs) collected from healthy human donors (ePBMCs; Cellular Technology Limited) were thawed and washed with ice cold Miltenyi MACS Buffer. NK cells were enriched from PBMCs using the Miltenyi NK Cell Isolation Kit (Miltenyi #130-092-657) following the manufacturer’s instructions. Isolated NK cells were counted and adjusted to a density of 1E6 cells/mL in assay media (RPMI-1640 media+ 5% ultra-low IgG FBS [Gibco #A3381901 + rhIL-2 100 U/mL [R&D Systems # 202-IL]). The primary NK cells (1E5 cells/well) were cultured in a 96-well V-bottom cell culture plate overnight at 37°C, 5% CO_2_ prior to use in stimulation assays.

### NK cell stimulation - IFNγ [ELISA]

In 96-well V-bottom cell culture plates, NK-92 or PTA-6967 cells (1E5/well) were incubated overnight at 37°C, 5% CO_2_ in NK cell media containing therapeutic protein products, polyclonal human IgG, or PMA (50 ng/mL) and ionomycin (1 µg/mL). For blocking studies, NK cells were pre-incubated with inhibitory molecules prior to the addition of 25nM rFVIIIFc. Following incubation, NK cells were pelleted, and cell culture supernatants were tested for human IFNγ levels by sandwich ELISA (BD #555142). Human IFNγ ELISA was performed as follows. Nunc MaxiSorp ELISA strips [ThermoFisher # 469949] were coated with purified anti-human IFNγ capture antibody [BD # 51-26131E] diluted in PBS (100 µL/well) of overnight at 4°C. Following overnight coating, wells were washed 3 times with wash buffer (PBS+0.05% Tween-20) and blocked with borate buffer (200 µL/well) for 1 hour at room temperature. A standard curve (2X dilution series) was prepared using a Recombinant Human IFN-γ Lyophilized Standard (BD # 51-26136E). Cell culture supernatant samples were prepared in borate buffer (1:5 dilution, 100 µL/well) and added to the ELISA plate for 1 hour at room temperature. Following sample incubation, wells were washed 5 times with wash buffer. A mixture of Detection Antibody Biotin Anti-Human IFN-γ [BD # 51-26132E] and Streptavidin-horseradish peroxidase conjugate (SAv-HRP) was prepared in 1:250 dilution in borate buffer and added to each well (100 µL/well) for 1 hour at room temperature. The wells were then washed 7 times with wash buffer (1 minute per wash). HRP enzyme substrate solution (tetramethylbenzidine (TMB); Surmodics # TMBS-0100-01) was added (100 µL/well) at room temperature and protected from light. After 20 minutes, stop solution (0.25 H_2_SO_4_, 50 µL/well) was added to each well. Absorbance at 450 nm was read using a BioTek ELx808 plate reader.

### NK cell stimulation - CD107a [flow cytometry]

Prior to stimulation, NK cell lines or isolated primary NK cells were washed with PBS. Cells were then resuspended in assay media (RPMI+5% UL IgG FBS) containing APC Mouse Anti-Human CD107a (BD #641581). Solutions of rFVIIIFc, BDD rFVIII, FL rFVIII, rFIXFc, KM33, GMA-8006, GMA-8013, GMA-8029, GMA-8014, or GMA-8002 were added to cells, and incubated at 37°C, 5% CO_2_ for 6 hours. After 1 hour of incubation, a mixture of Protein Transport Inhibitor Brefeldin A GolgiPlug (BD #555029) and Monensin GolgiStop (BD #554724) was added to the cells. After 6 hours post stimulation, cells were washed with PBS. Cells were then stained with viability marker (BD #564406) for 30 minutes on ice, washed with FACS buffer (PBS+1% FBS), and stained with FITC Mouse Anti-Human CD3 (BD #561806) and PECy7 Mouse Anti-Human CD56 (BD #560916) for 30 minutes on ice. Cells were then washed with FACS buffer, and then fixed (BD #554722) for 15 minutes on ice. Cells were then washed and resuspended with FACS buffer and stored at 4°C protected from light. Cells were analyzed by flow cytometry (BD LSRFortessa X20 Cell Analyzer).

PTA-6967 cells (1E6/well in 12 well plate; 1E5/well in 96 well plate) were incubated for 6 hours at 37°C, 5% CO2 in NK cell media containing 5 µL anti-CD107a-APC (BD #560664) as well as PMA (50 ng/mL) and ionomycin (1 µg/mL), or rFVIIIFc (250 nM). After one hour, monensin (0.67 µL/mL) and Brefeldin A (1 µL/mL) were added to block endocytic trafficking. Cell samples were harvested, washed, and stained for viability (Fixable Viability Dye eFluor506; eBioscience #65-2860) and surface markers [CD3-FITC (BD #561806), CD16-PE-Cy5 (BD #555408), CD56-PE-Cy5 (BD #557747)]. Samples were analyzed using a BD LSRII cytometer and FlowJo version 10 software.

### Biomolecular binding kinetics- biolayer interferometry

BLI biosensors [HIS1K (Sartorius # 18-5120)] were rehydrated in 1X kinetics buffer (PBS pH 7.4 supplemented with 0.02% Tween-20, 0.1% bovine serum albumin) for 10 minutes. All binding kinetics experiments were performed using the FortéBio Octet Red 96 machine with a total reaction volume of 1X kinetics buffer (200 µL/well). The kinetics assays included the following steps: (i) 60s initial baseline, (ii) 300s protein ligand immobilization, (iii) 60s baseline, (iv) 300s analyte association, and (v) 600s analyte dissociation. The protein ligand, CD16a-His [R&D Systems # 4325-FC] (250 ng/mL), was immobilized onto HIS1K biosensors. Analytes included rFVIIIFc, rFIXFc, IVIG, rituximab, and obinutuzumab [3200 - 50 nM or 400 - 6.25 nM concentration ranges]. Biomolecular binding data were acquired with Octet Data Acquisition software (release 8.2) and kinetics were calculated using a 1:1 fitting model with Octet Data Analysis software (release 8.2).

## Results

### rFVIIIFc potently induces CD16^+^ NK cell degranulation

**Figure 1:**
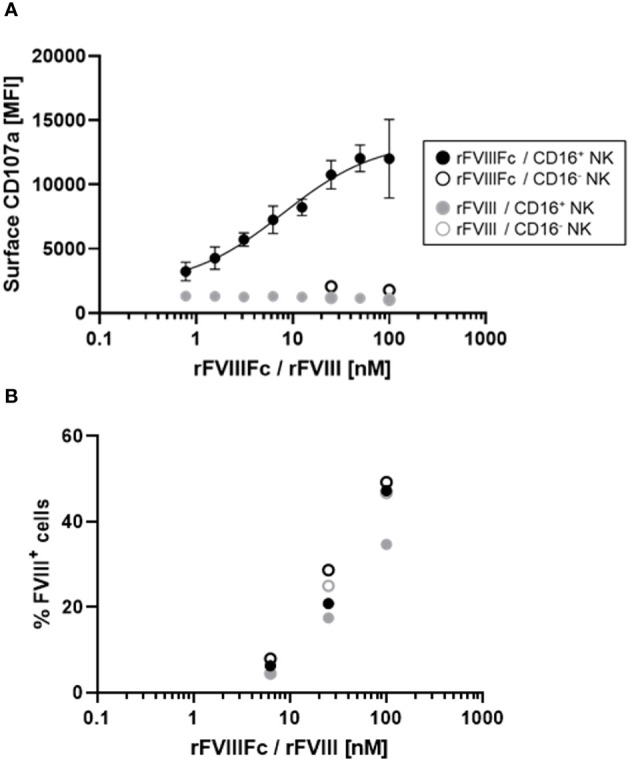
rFVIIIFc potently induces CD16^+^ NK cell degranulation. **(A)** Cell surface CD107a (lysosome-associated membrane protein 1, LAMP-1) mean fluorescence intensity levels measured using a flow cytometer following a six-hour cell culture incubation period. CD16^+^ NK cells (PTA-6967, filled circles) or CD16^-^ NK cells (NK-92, open circles) were incubated with different concentrations of rFVIIIFc (black circles) or rFVIII (grey circles). After one hour, monensin and brefeldin A were added to block endocytic trafficking and retain LAMP-1 on the plasma membrane surface. rFVIIIFc/CD16^+^ NK data from two independent experiments (n=4), all other data from a representative experiment (n=1). **(B)** Percentage of NK cells with surface-associated FVIII following a six-hour cell culture incubation period including protein transport inhibitors brefeldin A and monensin. Surface associated-FVIII was detected using a biotinylated anti-FVIII antibody and streptavidin-PE [rFVIIIFc (black circles) or rFVIII (grey circles), CD16^+^ NK cells (PTA-6967, filled circles) or CD16^-^ NK cells (NK-92, open circles)]. Data from representative experiment (n=1).

We previously demonstrated that CD16^+^ NK cells secrete the proinflammatory cytokine IFNγ and release cytolytic proteins (perforin and granzyme B) within hours of stimulation with rFVIIIFc (12). Here, we used a flow cytometry-based surface CD107a detection assay (20) to indirectly measure NK cell activation and degranulation. We compared surface CD107a levels on human CD16^+^ and CD16^-^ NK cell lines following a six-hour incubation with rFVIIIFc or FL rFVIII. Consistent with our prior findings, we measured potent, dose dependent rFVIIIFc-mediated CD16^+^ NK cell degranulation, with an EC_50_ of 7.9 nM (**Figure 1A**). Furthermore, rFVIIIFc specifically activated CD16/FcγRIIIA-expressing NK cells [PTA-6967], but not CD16^-^ NK cells [NK-92] (**Figure 1A**), demonstrating that Fc-CD16 interactions are necessary for dose-dependent rFVIIIFc-mediated NK cell activation. Importantly, rFVIII alone did not promote CD16^+^ NK cell degranulation.

Given the activation of CD16^+^ NK cells by rFVIIIFc, we hypothesized that rFVIIIFc may be bound to the NK cell surface and could be quantified. Thus, we assessed the percentage of FVIII^+^ NK cells by flow cytometry using a biotinylated anti-FVIII antibody. Following incubation of CD16^-^ or CD16^+^ NK cells with equivalent concentrations rFVIIIFc or rFVIII, we observed comparable percentages of FVIII^+^ NK cells (**Figure 1B**). However, only CD16^+^ NK cells incubated with rFVIIIFc resulted in NK cell degranulation. Thus, Fc-CD16 interactions are essential for activation of NK cells (**Figure 1**).

### rFVIIIFc mediates primary NK cell degranulation

**Figure 2:**
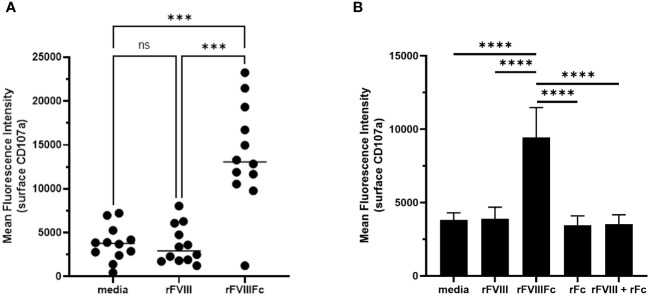
rFVIIIFc mediates primary NK cell degranulation. Cell surface CD107a (lysosome-associated membrane protein 1, LAMP-1) mean fluorescence intensity (MFI) levels measured using a flow cytometer following a six-hour cell culture incubation period including protein transport inhibitors brefeldin A and monensin. After one hour, monensin and brefeldin A were added to block endocytic trafficking and retain LAMP-1 on the plasma membrane surface. Each primary cell donor is represented by a single symbol which is the average of two data points. **(A)** Primary NK cells isolated from healthy human PBMC donors (n=12) were incubated with media alone, rFVIIIFc (25 nM), or rFVIII (25 nM). All data was collected in four independent experiments, each with cells isolated from three donors. Statistical comparison of means by one-way ANOVA and Tukey’s multiple comparisons test (*** = p < 0.001). **(B)** Primary NK cells isolated from healthy human PBMC donors (n=3) were incubated with media alone, rFVIIIFc (25 nM), rFVIII (25 nM), rFc (25 nM), or rFVIII (25 nM) + rFc (25 nM). Bars represent means with standard deviation of replicates (n=4) for each donor. Statistical comparison of means by one-way ANOVA and Tukey’s multiple comparisons test (**** = p < 0.0001).

We also used the flow cytometry-based detection of surface CD107a levels to test primary human NK cells for rFVIIIFc-mediated degranulation. Using NK cells isolated from cryopreserved PBMCs from healthy human donors, we found that rFVIIIFc robustly induced NK cell degranulation (**Figure 2A**). Using a single concentration of rFVIIIFc (25 nM), we measured a statistically significant increase in surface CD107a for NK cells (CD3^-^CD56^+^) incubated with rFVIIIFc, compared to media alone (p = 0.0001, ***) or rFVIII (25 nM) (p = 0.0002, ***) (**Figure 2A**). A statistically significant rFVIIIFc-mediated increase in surface CD107a levels (compared to media control and rFVIII stimulation) was observed for ten of the twelve donors tested. Importantly, an equimolar mixture of rFVIII with recombinant IgG1 Fc fragments (rFc) (25 nM) or rFc fragments alone (25 nM) did not induce elevated surface CD107a levels (**Figure 2B**), suggesting that covalent fusion between the FVIII and Fc moieties is required to elicit NK cell degranulation.

### Specificity of rFVIIIFc-mediated NK cell activation

**Figure 3:**
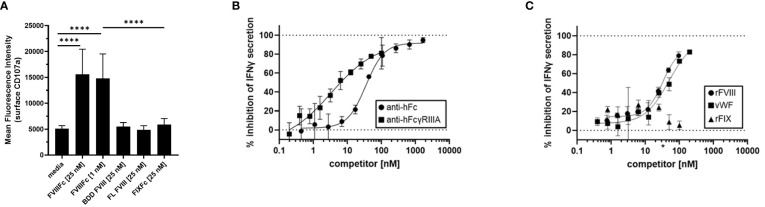
Specificity of rFVIIIFc-mediated NK cell activation. **(A)** Cell surface CD107a (lysosome-associated membrane protein 1, LAMP-1) mean fluorescence intensity (MFI) levels measured using a flow cytometer following a six-hour cell culture incubation period including protein transport inhibitors brefeldin A and monensin. Primary NK cells isolated from three healthy human PBMC donors were incubated with media alone, rFVIIIFc (25 nM, 1nM), B domain deleted (BDD) rFVIII (25 nM), full-length (FL) rFVIII (25 nM) or rFIXFc (25 nM). Bars represent means with standard deviation of replicates (n=2-6). All data was collected in three independent experiments, each with cells isolated from an individual donor. Statistical comparison of means by one-way ANOVA and Tukey’s multiple comparisons test (**** = p < 0.0001). **(B, C)** Secreted IFNγ levels in cell culture supernatant measured by ELISA following overnight incubation of CD16^+^ NK cells (PTA-6967) with rFVIIIFc (25 nM) and **(B)** goat anti-human Fc polyclonal antibody (circles) or mouse anti-human FcγRIIIA IgG1 (squares) or **(C)** rFVIII (circles), vWF (squares), or rFIX (triangles) as competitors for rFVIIIFc-mediated CD16^+^ NK cell activation. Inhibition is calculated as a percentage of secreted IFNγ level compared to no competitor control with dashed lines indicating a lack (0%) or complete (100%) inhibition. **(C)** * on x-axis indicates physiological vWF concentration, 35 nM. Data from representative experiment (n=1), each data point represents mean and standard deviation (n=3).

To demonstrate the specificity of rFVIIIFc-mediated NK cell activation, we tested related recombinant plasma protein products using primary NK cells isolated from healthy PBMC donors in the flow cytometry-based surface CD107a degranulation assay. We compared rFVIIIFc, BDD rFVIII, FL rFVIII, and rFIXFc. Consistent with results using human NK cell lines (**Figure 1**), primary human NK cells were activated and degranulated upon incubation with rFVIIIFc (**Figure 3A**) but did not respond to equimolar concentrations of either BDD rFVIII or FL rFVIII products.

To elucidate the role of each of the rFVIIIFc fusion protein domains on the activation of CD16^+^ NK cells, we targeted the FVIII and Fc moieties individually. As we have demonstrated that Fc-CD16 interactions are required for rFVIIIFc-mediated NK cell activation, we first investigated the consequence of disrupting the Fc domain and CD16/FcγRIIIA interactions. Pre-incubation of CD16^+^ NK cells with 3G8 (mouse anti-human CD16 IgG1 mAb) or a goat anti-human Fc polyclonal antibody resulted in potent inhibition [IC_50_, 3.3 nM and 34.8 nM respectively] of rFVIIIFc-mediated IFNγ secretion (**Figure 3B**). The anti-FcγRIIIA/CD16 and anti-Fc antibodies effectively disrupted rFVIIIFc-FcγRIIIA interactions and inhibited CD16^+^ NK pro-inflammatory cytokine secretion in a dose-dependent manner. We have shown above that rFVIIIFc activates NK cells while FVIII does not. We thus used a competition assay to demonstrate that rFVIII inhibited rFVIIIFc-mediated IFNγ secretion in a dose dependent manner [IC_50_, 32.7 nM] (**Figure 3C**). Furthermore, under physiological conditions, FVIII is bound to von Willebrand Factor (vWF) in human blood [vWF : FVIII, binding affinity, 0.2 nM (21)]. Pre-incubation of rFVIII with vWF also resulted in the inhibition of rFVIIIFc-mediated IFNγ secretion [IC_50_, 49.6 nM]. However, FIX (which does not bind to FVIII in its non-activated form) did not result in rFVIIIFc-mediated IFNγ secretion following pre-incubation (**Figure 3C**).

### FVIII LC involved in rFVIIIFc-mediated CD16^+^ NK cell activation

**Figure 4:**
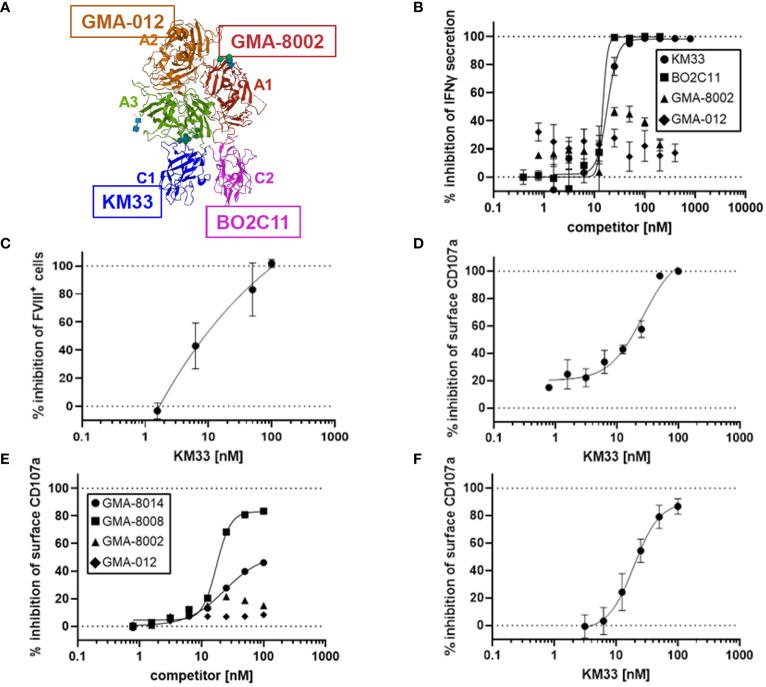
FVIII LC involved in rFVIIIFc-mediated CD16^+^ NK cell activation. **(A)** Structure of FVIII component of rFVIIIFc (PDB: 5K8D). FVIII domains are labeled and colored individually. FVIII-specific antibodies used in **Figure 4B** are highlighted: anti-A1 GMA-8002 (red), anti-A2 GMA-012 (orange), anti-C1 KM33 (blue), and anti-C2 BO2C11 (magenta). **(B)** Secreted IFNγ levels in cell culture supernatant measured by ELISA following overnight incubation of CD16^+^ NK cells (PTA-6967) with rFVIIIFc (25 nM) and anti-FVIII binding KM33 (circles), BO2C11 (squares), GMA-8002 (triangles), or GMA-012 (diamonds) as competitors for rFVIIIFc-mediated CD16^+^ NK cell activation. Inhibition is calculated as a percentage of secreted IFNγ level compared to no competitor control with dashed lines indicating a lack (0%) or complete (100%) inhibition. Each data point represents mean and standard deviation (n=3) from a representative experiment. **(C)** Percentage of CD16^+^ NK cells (PTA-6967) with surface-associated FVIII following a six-hour cell culture incubation period with 25 nM rFVIIIFc and KM33 as competitor including protein transport inhibitors brefeldin A and monensin. Surface associated-FVIII was detected using a biotinylated anti-FVIII antibody and streptavidin-PE. Inhibition is calculated as a percentage of FVIII^+^ cells compared to no competitor control with dashed lines indicating a lack (0%) or complete (100%) inhibition. Each data point represents mean and standard deviation (n=3) from a representative experiment. **(D)** Cell surface CD107a (lysosome-associated membrane protein 1, LAMP-1) mean fluorescence intensity (MFI) levels measured using a flow cytometer following a six-hour cell culture incubation period including protein transport inhibitors brefeldin A and monensin. CD16^+^ NK cells (PTA-6967) were incubated with rFVIIIFc (25 nM) and anti-FVIII KM33 (circles) as a competitor for rFVIIIFc-mediated CD16^+^ NK cell activation. Each data point represents a mean with standard deviation of replicates (n=2) from a representative experiment. **(D-F)** Inhibition is calculated as a percentage of surface CD107a MFI level compared to no competitor control with dashed lines indicating a lack (0%) or complete (100%) inhibition. **(E, F)** Cell surface CD107a (lysosome-associated membrane protein 1, LAMP-1) mean fluorescence intensity (MFI) levels measured using a flow cytometer following a six-hour cell culture incubation period including protein transport inhibitors brefeldin A and monensin. **(E)** CD16^+^ NK cells (PTA-6967) were incubated with 25 nM rFVIIIFc and anti-FVIII antibodies as competitors [GMA-8008 (squares), GMA-8014 (circles), GMA-8002 (triangles), GMA-012 (diamonds)] of rFVIIIFc-mediated CD16^+^ NK cell activation, each symbol represents a single datapoint (n=1) from a representative experiment. **(F)** Primary NK cells isolated from a single healthy human PBMC donor were incubated with 25 nM rFVIIIFc and anti-FVIII KM33 (circles) as a competitor of rFVIIIFc-mediated CD16^+^ NK cell activation, each symbol represents the mean and standard deviation (n=3) from a representative experiment.

Given vWF inhibition of rFVIIIFc-mediated CD16^+^ NK cell activation (**Figure 3C**) and FVIII LC domains responsible for binding to vWF (21), we tested the role of different FVIII domains in rFVIIIFc-mediated CD16^+^ NK cell activation. We used domain-specific anti-FVIII antibodies to assess the role of the FVIII LC and HC domains in NK cell activation (**Figure 4A**). We demonstrated that FVIII LC-specific antibodies (KM33, anti-FVIII C1 domain scFv; BO2C11, anti-FVIII C2 domain hIgG4) inhibited rFVIIIFc-mediated IFNγ secretion from CD16^+^ NK cells (**Figure 4B**). However, FVIII HC-specific antibodies (GMA-8002, anti-FVIII A1 domain mIgG; GMA-012, anti-FVIII A2 domain mIgG) did not inhibit CD16^+^ NK pro-inflammatory cytokine secretion (**Figure 4B**). Anti-FVIII C1 domain scFv (KM33) decreased surface-bound FVIII levels (**Figure 4C**) and potently inhibited rFVIIIFc-mediated CD16^+^ NK cell degranulation [IC_50_, 26.5 nM] (**Figure 4D**). Similarly, additional anti-FVIII C2 domain-specific antibodies (GMA-8008 and GMA-8014) inhibited rFVIIIFc-mediated CD16^+^ NK cell degranulation, while FVIII HC-specific antibodies did not (**Figure 4E**). We found that the role of the FVIII domain C1 in rFVIIIFc-mediated NK cell activation remained consistent across different NK cell sources, as we observed a similar inhibitory effect using KM33 and primary NK cells isolated from an individual healthy PBMC donor [IC_50_, 19.5 nM] (**Figure 4F**) as using KM33 and the human CD16^+^ cell line (**Figure 4D**). The findings that both the C1 and C2 domains of FVIII LC are involved can be explained by the proximity of these two domains on the FVIII molecule as depicted in **Figure 4A**.

### CD16 binding kinetics

We compared the binding kinetics of rFVIIIFc for CD16 with other Fc-containing therapeutic protein products using bio-layer interferometry (BLI) in a cell free system using the purified CD16 protein. The association rates (K_a_), dissociation rates (K_d_), and binding affinities (K_D_) are summarized in **Table 1**. Therapeutic mAbs rituximab and obinutuzumab were included as human IgG1 Fc-containing controls. We found that rFVIIIFc and obinutuzumab (afucosylated) have similar Fc-mediated binding kinetics for CD16, but observed differences in the binding kinetics of rFVIIIFc and rFIXFc Fc-fusion proteins (**Table 1**). It is important to note that the binding parameters (on-rate and off-rate) for rFIXFc are more variable compared to those obtained for rFVIIIFc. Importantly, the binding affinity (K_D_) of rFVIIIFc was about 5-fold higher than that of rFIXFc.

**Table 1:** CD16:Fc binding kinetics.

analyte	K_D_ [M]	k_a_ [1/Ms]	k_d_ [1/s]	curve fit R^2^
IVIG	2.436e-007 [1.153e-006 - 5.148e-008]	165132 [28126 – 969527]	0.04024 [0.02671 - 0.06062]	0.9930 [0.9876 - 0.9985]
rituximab	2.614e-007 [4.841e-007 - 1.412e-007]	844562 [359523 – 1983976]	0.2207 [0.1219 - 0.3996]	0.9932 [0.9886 - 0.9978]
obinutuzumab	4.532e-008 [6.763e-008 - 3.036e-008]	434793 [338173 – 559020]	0.01970 [0.01591 - 0.02440]	0.9945 [0.9905 - 0.9986]
rFVIIIFc	5.504e-008 [1.170e-007 - 2.588e-008]	358052 [228726 – 560502]	0.01970 [0.01423 - 0.02729]	0.9896 [0.9790 - 1.000]
rFIXFc	2.816e-007 [7.855e-006 - 1.009e-008]	27995 [3902 – 200842]	0.007882 [0.0006237 - 0.09960]	0.9813 [0.9699 - 0.9927]

Biomolecular binding kinetics of analytes [IVIG, rituximab, obinutuzumab, rFVIIIFc, and rFIXFc] to CD16a protein immobilized to a biosensor surface. Mean binding kinetics values include the affinity [equilibrium binding constant (K_D_)], on-rate [association binding constant (k_a_)], and off-rate [dissociation rate (k_d_)]. Mean curve fit values (R^2^) above 0.95 indicate a good correlation between the experimental data and the fitting model (Octet Data Analysis software release 8.2). For each analyte, the geometric mean binding kinetics or curve fit value represents datapoints of 5-11 analyte concentrations from one or two independent experiments. For each geometric mean value, the 95% confidence interval is indicated in brackets.

### The role of LRP1 and plasma membrane lipid binding in rFVIIIFc-mediated CD16^+^ NK cell activation

**Figure 5:**
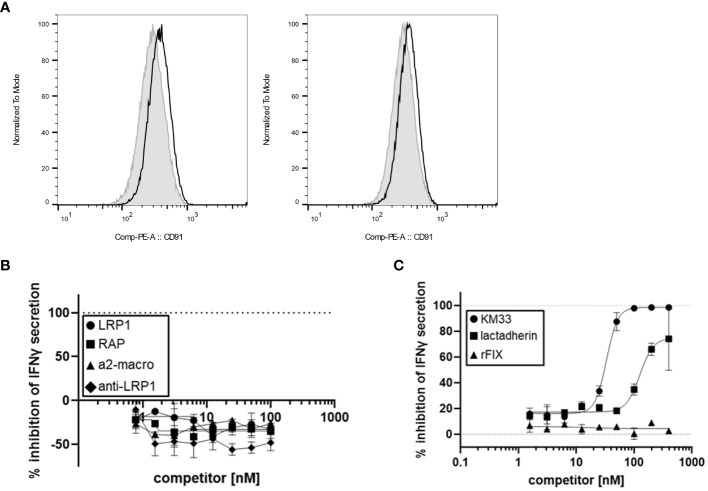
FVIII:plasma membrane interactions contribute to rFVIIIFc-mediated CD16^+^ NK cell activation. **(A)** Histograms of CD91 (LRP1) mean fluorescence intensity (MFI) levels on the surface of CD16^-^ NK cells (NK-92, left) and CD16^+^ NK cells (PTA-6967, right). The grey shaded histogram represents unstained cells, and the black histogram represents cells stained with anti-human CD91-PE. **(B, C)** Secreted IFNγ levels in cell culture supernatant measured by ELISA following overnight incubation of CD16^+^ NK cells (PTA-6967) with rFVIIIFc (25 nM) and **(B)** LRP1 (circles) or LRP1 ligands [RAP (squares), a2-macroglobulin (triangles), anti-LRP1 IgG (diamonds)] or **(C)** KM33 (circles), lactadherin (squares), or rFIX (triangles) as competitors for rFVIIIFc-mediated CD16^+^ NK cell activation. **(B, C)** Inhibition is calculated as a percentage of secreted IFNγ level compared to no competitor control with dashed lines indicating a lack (0%) or complete (100%) inhibition. Data from representative experiment (n=1), each data point represents mean and standard deviation (**(B)**, n=2; **(C)**, n=2-3).

Based on our finding that FVIII LC plays a role in rFVIIIFc-mediated CD16^+^ NK cell activation, we hypothesized the FVIII moiety may interact with cell surface molecules. Low-density lipoprotein (LDL) receptor-related protein 1 (LRP1) is a cell surface receptor that functions as a FVIII clearance receptor by binding to the LC domains when FVIII is not bound to vWF (22). Given the importance of FVIII-LRP1 interactions, we investigated the role of LRP1 binding in rFVIIIFc-mediated CD16^+^ NK cell activation. However, flow cytometric analysis demonstrated that CD16^+^ (PTA-6967) and CD16^-^ (NK-92) NK cell lines do not express appreciable surface levels of LRP1/CD91 (**Figure 5A**). Furthermore, using LRP1, an anti-LRP1 binding antibody, and soluble LRP1 ligands (RAP and a2-macroglobulin) as competitors we also demonstrated that LRP1 blockade does not inhibit rFVIIIFc-mediated activation of CD16^+^ NK cells (**Figure 5B**). Our results suggest LRP1 does not serve as a key FVIII LC receptor facilitating rFVIIIFc-mediated NK cell activation. Thus, it is likely the rFVIIIFc fusion protein FVIII LC domains may interact with other cell surface FVIII receptor proteins or plasma membrane lipids.

Lactadherin is a phospholipid-binding multi-functional protein secreted by mammary epithelial cells and macrophages that is abundant in mammalian breast milk and promotes phagocytosis of apoptotic cells (23, 24). Lactadherin contains two C-terminal domains that share sequence and structural homology to the FVIII C1 and C2 domains (23, 25). Previously, lactadherin has been shown to compete with FVIII for binding to phosphatidylserine-containing membranes (23). Thus, we hypothesized that lactadherin could compete with rFVIIIFc for attachment to the NK cell plasma membrane surface, and we assessed whether phospholipid-binding contributes to rFVIIIFc-mediated CD16^+^ NK cell activation. Competition of rFVIIIFc-mediated proinflammatory cytokine secretion by lactadherin [**Figure 5C**] suggests a role for FVIII-mediated phospholipid binding in CD16^+^ NK cell activation.

## Discussion

Functional characterization of the rFVIIIFc fusion protein through *in vitro* studies, as described here and previously by our group (12) and others (9–11, 13), have shown diverse Fc-mediated outcomes with respect to the immune system. These studies have demonstrated that rFVIIIFc interactions result in a wide range of Fc effector functions including activation of CD16^+^ NK cells (12), induction of regulatory macrophage polarization (9), inhibition of B-cell activation (10), activation of dendritic cells (DCs) (11), and inhibition of CD14^+^ blood monocyte differentiation into osteoclasts (13). We previously demonstrated the NK cell lines used in this study (NK-92 and PTA-6967) do not express high-affinity FcγRI/CD64 or low-affinity FcγRII/CD32 receptors (12), enabling us to elucidate the role of rFVIIIFc on FcγRIIIA/CD16-mediated cellular activation. We observed that rFIXFc, a fusion protein sharing an identical IgG1 Fc domain amino acid sequence as rFVIIIFc (26–28), did not activate CD16^+^ NK cells (**Figure 3A**). Thus, we hypothesized that the FVIII moiety of rFVIIIFc may play a role in CD16^+^ NK cell activation. Herein we have demonstrated that the FVIII LC domains influence the rFVIIIFc Fc effector function.

We observed that rFVIIIFc functions differently, with respect to the Fc effector function, when compared to other IgG1 Fc-containing therapeutic proteins (29). We have demonstrated the rFVIIIFc fusion protein potently activated CD16^+^ NK cells in the absence of other cell types (i.e., target cells), which, in the case of many therapeutic IgG1 mAbs, is generally a prerequisite for CD16^+^ NK cell activation and the antibody-dependent cellular cytotoxicity (ADCC) mechanism of action due to FcγR crosslinking and avidity effects. In the context of mAbs, there is evidence that distal regions/domains of the same molecule, such as the antigen-binding Fab region, contribute to stabilizing Fc-FcγRIII interactions (30–32) and impact Fc effector function. Furthermore, antigen binding by mAbs can also impact Fc-FcγR interactions (33, 34). In the case of Fc-fusion proteins, the Fc domain is covalently linked to a bioactive fusion partner, therefore, it would not be unreasonable to speculate that the bioactive fusion partner’s biology which includes binding to other proteins, lipids, or carbohydrate molecules may influence Fc binding and functional properties.

FVIII and FIX are plasma proteins with distinct structural and functional properties that are integral to maintaining hemostasis as part of the blood coagulation cascade. The FVIII zymogen circulates as a heterodimeric protein consisting of a HC made up of A1 and A2 domains, a B domain, and the LC made up of A3, C1, and C2 domains. Following proteolytic cleavage and activation by thrombin or activated Factor X (FXa), activated FVIII (FVIIIa) acts as an enzyme cofactor by forming a complex with activated Factor IX (FIXa) in the presence of Ca^2+^ and phospholipids. The FIX zymogen circulates as a multi-domain protein consisting of a gamma-carboxyglutamic acid (Gla) domain, two epidermal growth factor domains, and a trypsin-like peptidase domain. FIXa, a serine protease in complex with FVIIIa, cleaves and activates Factor X. Importantly, the FVIII LC C1 and C2 moieties contain discoidin-like domains (35). The FVIII C1 and C2 domains belong to a family of discoidin protein domains, which share similarity to the discoidin lectin of *Dictyostelium discoideum* (a slime mold) (36, 37). The *D. discoideum* discoidin lectin binds to galactose and promotes binding to anionic phospholipids (36). Mammalian proteins including coagulation factor V, factor VIII, and lactadherin each contain discoidin-like domains that form membrane-binding β-barrel structures (38–40). Biochemical studies have elucidated the role of the FVIII C1 and C2 domains in membrane phospholipid binding (41–43).

Here (**Figure 3A**) and previously (12), we have observed differences between rFVIIIFc and rFIXFc with respect to activation of NK cells despite shared human IgG1 Fc domain amino acid sequences (26–28). In sharp contrast to rFVIIIFc, rFIXFc did not activate primary human NK cells (**Figure 3A**). We hypothesized differences in the structure and function of FVIII and FIX likely account for differences in Fc effector function and CD16^+^ NK cell activation potential. Specifically, we demonstrated FVIII bound the NK cell surface (**Figure 1B**) but was not sufficient to activate NK cells (**Figures 1A**, **2A**). Furthermore, pre-incubation of NK cells with rFVIII inhibited rFVIIIFc-mediated cellular activation while pre-incubation with rFIX did not (**Figure 3C**), suggesting direct competition of NK cell surface binding sites by rFVIII. However, it is important to note that in our *in vitro* experiments we have used inactivated forms of rFVIII, rFVIIIFc, rFIX, and rFIXFc, thus the results may not fully reflect the functional properties of the activated forms. With this limitation, we investigated the role of the FVIII moiety with respect to the rFVIIIFc-mediated activation of NK cells. Adding antibodies specific to different domains of FVIII as inhibitors of NK cell activation revealed that the C1 and C2 domains of the FVIII LC were involved in NK cell activation, but the A1 and A2 HC domains were not.

Subsequently, we demonstrated that LRP1 may not be the major cell surface receptor for FVIII on NK cells (**Figures 5A, B**). However, we did show that lactadherin, a phospholipid-binding protein containing discoidin domains similar to FVIII C1 and C2 domains, did compete with rFVIIIFc binding to the plasma membrane and prevent NK cell activation (**Figure 5C**) suggesting that FVIII-mediated phospholipid-binding may promote Fc-CD16 interactions.

Our findings implicating FVIII LC domains in rFVIIIFc Fc effector function (**Figure 4**) are consistent with recent findings by Duan et al. (13), demonstrating that rFVIIIFc inhibits CD14^+^ blood monocyte differentiation into osteoclasts via Fc-FcγRII interactions on the monocyte surface. Moreover, Duan et al., demonstrated that rFVIIIFc-mediated inhibition of monocyte-derived osteoclast formation is dependent on FVIII C1 and C2 domain binding on the monocyte surface, described as a “double touchpoint” model. Interestingly, FVIII C1 and C2 domains have also been linked to poor transplacental delivery of rFVIIIFc in mouse models (44) suggesting the rFVIIIFc FVIII LC domains may also impact Fc interactions with the neonatal Fc receptor (FcRn), responsible for transcytosis of IgG across syncytiotrophoblasts and transplacental delivery of maternal IgG to the fetus (45). While the exact nature of rFVIIIFc and FcRn interactions as related to transplacental transfer are still unknown, rFVIIIFc-FcRn interactions do confer an enhancement in circulating half-life as compared to non-Fc-fused FVIII products, demonstrated in animal models (46) and in clinical trials (47, 48).

Interestingly, while we have demonstrated that the FVIII LC domains play a role in rFVIIIFc-mediated CD16^+^ NK cell activation, our measurements of binding kinetics suggest that the FVIII moiety impacts Fc binding properties even in the absence of a cell surface. Using a cell-free soluble binding system, which would negate FVIII C1 and C2-mediated cell surface binding, we observed differences in binding kinetics between rFVIIIFc and rFIXFc (**Table 1**), with rFVIIIFc having similar FcγRIIIA binding kinetics as obinutuzumab, an afucosylated IgG1 mAb glyco-engineered for enhanced engagement with CD16 (49). These data suggest that the FVIII moiety may not only provide a means for attachment to the cell surface providing avidity and allowing monomeric Fc to overcome the low-affinity FcγR activation threshold, but the presence of the FVIII moiety also likely affects the Fc domain’s affinity for CD16. This finding is consistent with our earlier finding that Fc-FcγR and Fc-complement C1q binding and signaling properties are influenced by the active molecular entity covalently attached to the Fc in different fusion proteins (29). This may help explain rFVIIIFc’s pleotropic effects on many different FcγR-expressing cell types in *in vitro* studies. Interestingly we observed that rFVIIIFc binds CD16 with a ~5-fold higher affinity than rFIXFc, attributable to differences in both the association and dissociation rate constants (**Table 1**). These results could be explained by our previous findings (29) demonstrating that Fc-Fc receptor interactions are influenced by the molecular entity that is fused to the Fc fragment. This is also consistent with observations that different Fc-fusion proteins have different serum half-lives (6). Similarly, in general, the half-life of Fc-fusion proteins is considerably lower than that of antibodies. Taken together these *in vitro* data and preclinical and clinical observations suggest that allosteric effects attributable to the ‘fusion-partner’ affect the Fc binding kinetics.

**Figure 6:**
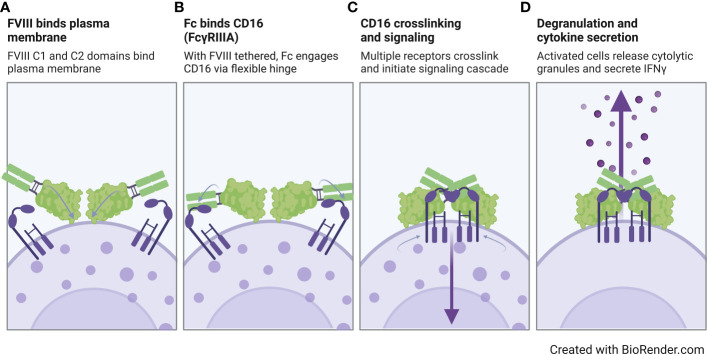
Proposed model of rFVIIIFc interactions with CD16^+^ NK cells. Illustration of a proposed model for rFVIIIFc interactions with CD16^+^ NK cells highlighting key molecular events including **(A)** FVIII binding to the NK cell plasma membrane, **(B)** fusion protein Fc domain engaging with CD16, **(C)** multiple Fc : CD16 interactions resulting in receptor crosslinking and intracellular signaling, and **(D)** activation of NK cell leading to degranulation and inflammatory cytokine secretion. Image created with BioRender.com.

Overall, our findings suggest that the FVIII moiety plays a role in rFVIIIFc CD16 signaling, but FVIII alone is not sufficient for pro-inflammatory NK cell activation. In **Figure 6**, we propose a model for rFVIIIFc interactions with CD16^+^ NK cells. In our proposed model, we hypothesize (i) the rFVIIIFc FVIII C1 and C2 domains bind the plasma membrane; (ii) with the FVIII moiety tethered, the flexible hinge region promotes enhanced engagement of a high affinity Fc domain with CD16; (iii) upon crosslinking of multiple CD16 receptors, the intracellular signaling cascade is initiated; and (iv) resulting in NK cell activation, including release of pre-formed cytolytic granules (degranulation) and pro-inflammatory cytokine secretion (IFNγ). Our proposed model is supported by a detailed rFVIIIFc structural study demonstrating the flexibility and rotational freedom at the fusion site (Fc hinge region) and functional independence between the rFVIII and Fc moieties (50).

While the pleotropic effects of rFVIIIFc have been demonstrated in multiple *in vitro* studies and associated with multiple FcγR-expressing leukocyte populations including monocytes, macrophages, DCs, B cells, and NK cells (9–13), our data may provide a plausible molecular mechanism underlying the potential of using rFVIIIFc in ITI therapy to eradicate inhibitors as described in retrospective case reports (16–18) and a prospective ITI study (verITI-8) (19). However, we acknowledge that *in vitro* studies have limitations, and do not claim the observations reported herein fully capture the complexity of *in vivo* systems or the clinical experience of patients with hemophilia A treated with rFVIIIFc. Consistent with other *in vitro* studies characterizing the Fc effector function of rFVIIIFc, we measured NK cell activation using hyper-physiological FVIII concentrations, which do not correlate with circulating rFVIIIFc levels during standard prophylaxis treatment. However, the concentrations we used *in vitro* may be more relevant to the use of rFVIIIFc during high-dose ITI regimens (Bonn protocol). In the ITI regimen patients with hemophilia A and high titer inhibitors are administered daily doses of rFVIIIFc at 200 IU/kg. Additionally, in circulation, there is likely to be limited accessibility to the FVIII C1 and C2 domains, as FVIII forms a noncovalent complex with vWF (see **Figure 3C** for vWF-mediated inhibition). Based on the *in vitro* data, *in vivo* vWF could considerably inhibit rFVIIIFc from activating CD16^+^ NK cells at the physiological vWF concentration (~35 nM) (21), however, higher rFVIIIFc dosing levels during ITI therapy may exceed the vWF inhibitory threshold and allow for rFVIIIFc Fc-mediated immunomodulatory properties. Of course, *in vitro* studies are unable to model the biological outcomes in circulation where rFVIIIFc can interact with multiple FcγR-expressing immune cell types at the same time.

More broadly, Fc-fusion proteins have previously been designed to act specifically as antigen-specific B cell depletion agents. For example, Fc fusion proteins with human myelin oligodendrocyte glycoprotein (MOG) or a myelin basic protein immunodominant peptide have both been shown to specifically deplete myelin-reactive B cells using *in vivo* animal models (51, 52). These studies provide proof-of-concept for the use of Fc-fusion proteins as antigen-specific B cell depletion agents. Perhaps novel Fc fusion proteins can be designed using Fc domains engineered with enhanced affinity to CD16 to create potent antigen-specific B cell depletion agents. One could speculate that such a fusion protein may have clinical utility in the case of autoimmune disorders with a well-characterized antigen or in the case of immunogenic therapeutic protein product (i.e., FVIII for hemophilia A or recombinant acid-alpha glucosidase (rGAA) for Pompe’s disease) as the target. Such a Fc-fusion B cell depletion agent would target B cell receptor (BCR)-expressing memory B cells and would provide a more targeted B cell depletion therapy strategy than anti-CD20 targeting monoclonal antibodies (including rituximab, obinutuzumab, ocrelizumab, ofatumumab, and ublituximab), which deplete the entire CD20^+^ B cell population. While these mAbs have successfully been used for the treatment of autoimmune diseases (53) and B-cell malignancies (54), these global B-cell depletion therapy strategies put the patient at increased risk of infection. We previously demonstrated that rFVIIIFc promotes CD16^+^ NK cell-mediated lysis of a FVIII-specific B cell clone BO2C11 (12). Therefore, an antigen-specific B cell depletion strategy, such as a CD16^+^ NK cell activating Fc-fusion protein, could provide a favorable safety benefit.

## Data availability statement

The original contributions presented in the study are included in the article/supplementary material, further inquiries can be directed to the corresponding author/s.

## Ethics statement

Ethical approval was not required for the studies on humans in accordance with the local legislation and institutional requirements because only commercially available cryopreserved peripheral blood mononuclear cells and established cell lines were used.

## Author contributions

HL: Conceptualization, Formal analysis, Investigation, Writing – original draft, Writing – review & editing. JO: Formal analysis, Investigation, Writing – original draft. ZS: Conceptualization, Formal analysis, Funding acquisition, Supervision, Writing – review & editing. BG: Conceptualization, Formal analysis, Funding acquisition, Supervision, Writing – review & editing.
